# Lactose-free milk prolonged endurance capacity in lactose intolerant Asian males

**DOI:** 10.1186/s12970-014-0049-4

**Published:** 2014-10-23

**Authors:** Kriyot Sudsa-ard, Kallaya Kijboonchoo, Visith Chavasit, Rungchai Chaunchaiyakul, Amanda Qing Xia Nio, Jason Kai Wei Lee

**Affiliations:** Institute of Nutrition, Mahidol University, Salaya, Phutthamonthon, Nakhon Pathom 73170 Thailand; College of Sports Science and Technology, Mahidol University, Salaya, Phutthamonthon, Nakhon Pathom 73170 Thailand; School of Sport, Cardiff Metropolitan University, Cardiff, UK; Defence Medical and Environmental Research Institute, DSO National Laboratories, Singapore, Singapore; Yong Loo Lin School of Medicine, National University of Singapore, Singapore, Singapore; Lee Kong Chian School of Medicine, Nanyang Technological University, Singapore, Singapore

**Keywords:** Lactose intolerance, Hydration, Recovery drink, Cycling, Endurance capacity, Asian population

## Abstract

**Background:**

Several studies on Caucasian volunteers have proven that milk is an effective recovery drink for athletes. Such benefit, however, cannot be directly applied to the lactose-intolerant Asian population. This study investigated the effects of ingesting water (WT), sports drink (SPD) and lactose-free milk (LFM) on cycling capacity.

**Methods:**

Ten healthy young men completed 3 randomized experimental trials. Each trial consisted of an intermittent glycogen depleting session, a 2 h recovery period during which they ingested the test drink, followed by cycling at 70% of their maximum oxygen consumption (VO_2_max) to volitional exhaustion. Each trial was separated by at least one week.

**Results:**

There were no complaints or symptoms of lactose intolerance during any of the trials. The cycling periods were different (p < 0.05) amongst the 3 trials, namely, lactose-free milk (LFM; 69.6 ± 14.0 min), sports drink (SPD; 52.1 ± 11.6 min), and water (WT; 36.0 ± 11.1 min), respectively. The VO_2_ and VCO_2_ of LFM (30 ± 4 and 29 ± 4 ml/kg/min) were lower (p < 0.05) than that of SPD (34 ± 4 and 34 ± 4 ml/kg/min) and WT (35 ± 4 and 33 ± 5 ml/kg/min). There were no differences (p = 0.45) in VO_2_ and VCO_2_ between SPD and WT. Mean rating of perceived exertion was lowest in LFM (14 ± 5; p < 0.05), while no difference was found between the other two trials (SPD: 16 ± 4 and WT: 16 ± 4; p = 0.18).

**Conclusion:**

Lactose-free milk is likely to be an effective recovery drink for enhancing subsequent cycling capacity in lactose intolerant Asian males.

## Introduction

Nutrition is one aspect of an athlete’s lifestyle that can be modified to enhance sporting performance. This encompasses individualized interventions adopted before, during, and after exercise. Appropriate nutrition after exercise can enhance recovery and augment performance in the subsequent exercise bout, which may in turn, encourage greater physiological adaptation to exercise training and result in improved performance during competition.

Milk has been proven to be an effective post-exercise drink for endurance activities [1-7]. Milk contains several ergogenic nutrients including carbohydrate, protein, fat, vitamins, and minerals. Following an exhaustive bout of exercise, athletes who drank milk could recover faster and exhibit better exercise performance compared with those who had commercial sports drinks or carbohydrate replacement drink [5,6]. Moreover, an electrolyte drink that was fortified with carbohydrate and protein could increase muscle glycogen by 128% more than a 6% carbohydrate drink [8]. Beradi et al. [9] explained that muscle glycogen resynthesis was greater following 6 h of recovery due to the addition of protein to the recovery drink.

Unfortunately, the benefits of protein and carbohydrate, especially from milk, in enhancing the recovery period of athletes are not applicable to lactose intolerance individuals, such as Asians. Symptoms of lactose intolerance can include nausea, vomiting, and diarrhea, with the severity of symptoms dependent on the level of lactose intolerance. Such an effect will likely impair exercise performance. A study performed in indigenous Asians in Singapore showed that all of the sampled 22 subjects aged 15–42 y were lactose-intolerant [10]. Asmawi et al. [11] reported hypolactasia in 88% of Malaysian Malays, 91% of Malaysian Chinese, and 83% of Malaysian Indians. A study using the breath-hydrogen test after oral intake of 25 g lactose in Thai adults demonstrated that almost half of the cohort was lactose intolerant [12].

Lactose-free milk (LFM) may be potentially ergogenic as a recovery beverage for lactose intolerant individuals. We hypothesized that a LFM drink could extend cycling time to exhaustion. To our knowledge, there is no study of LFM on prolonged exercise. We investigated the effects of LFM on endurance cycling capacity in healthy Thai males.

## Methods

### Study participants

Ten healthy males volunteered for this study (Table 1). Experimental procedures were approved by Mahidol University Institutional Review Board, Thailand. Participants received a verbal explanation about the study before providing written informed consent.

**Table 1:** **Physical and physiological characteristics of the participants (n = 10)**

	**Mean ± SD**	**Range**
Age (yr)	21.2 ± 0.8	20–22
Height (cm)	174 ± 3	168–178
Body weight (kg)	66.8 ± 4.6	60.8–73.8
BMI (kg/m^2^)	22.1 ± 1.5	20.3–24.8
Body fat (%)	12.6 ± 4.3	9.7–19.7
VO_2_max (ml/kg/min)	44 ± 2	40–47
Peak power output (W)	288 ± 41	280–320

### Laboratory protocol

Participants arrived at the laboratory in the morning following an overnight fast. Each participant completed four visits to the laboratory (ambient temperature: 25 ± 1°C; relative humidity: 56% ± 2): a maximal incremental exercise test and three randomized experimental trials ingesting water (WT), a commercial sports drink (SPD), or lactose-free milk (LFM; manufactured by the Institute of Nutrition, Mahidol University, Thailand; Table 2).

**Table 2:** **Nutritional content in 250 mL of each test drink**

**Contents**	**Water**	**Sports drink**	**Lactose-free milk**
Energy (kcal)	-	100	100
Carbohydrate (g)	-	25.0	12.5
Protein (g)	-	-	8
Fat (g)	-	-	2
Sodium (g)	-	0.092	0.439
Potassium (g)	-	0.015	0.518
Osmolality (mosmol/kg)	-	415	352

Lactose-free milk was produced by adding 500 ppm β-galactosidase (lactase) enzyme (Ha-Lactase 5200 NLU/g, Chr-Hansen, Horsholm, Denmark) to an in-house low-fat pasteurized milk. The inoculated milk was incubated at 8°C for 24 h before pasteurization at 72°C for 15 s. The pasteurized milk was cooled to room temperature and fortified with sodium and potassium to increase its osmolality which was initially much lower than the sports drink.

The experimental trial consisted of an exercise-induced glycogen depletion session, a 2-h recovery period during which the test drink was ingested, and an endurance capacity test. Each trial was separated by 1–2 weeks. Dietary intake in the three days prior to the first experimental trial was recorded and repeated for subsequent trials. The macronutrients ingested were calculated using an in-house dietary software programme (INMUCAL, Institute of Nutrition, Mahidol University, Thailand). Urine specific gravity (Refractometer model 300CL, Atago Inc, Japan) was recorded as a measure of hydration status before each experimental trial.

### Maximal incremental exercise test

Participants completed a standardized 2-min warm-up (cadence 60 revolutions [rev]/min, workload 0.5kp) on a cycle ergometer (Ergomedic 828 E, Sweden) followed by an incremental cycling test to volitional exhaustion at a cadence of 80 rev/min. Workload was increased by 1 kp every 2 min. Oxygen uptake was determined using a metabolic cart (Vmax Sensor Medics Metabolic, SensorMedics® Corporation, USA). Heart rate (Polar RS800CX POLAR®, Finland) was measured. The test was accepted if at least two of following criteria were met: i) respiratory exchange ratio (RER) greater than 1.1; ii) heart rate above 90% of age-predicted maximum heart rate; and iii) a VO_2_ increase of less than 0.15 l/min from the previous workload [13]. Maximum power output (Pmax) was defined as the power output attained during the final completed stage.

### Glycogen depletion session

The glycogen depletion exercise consisted of 2-min intervals at 60%–90%Pmax interspersed with 50%Pmax recovery at 80 rev/min [5]. Participants commenced the exercise with 2-min intervals at 90%Pmax and the workload was subsequently reduced to 80%Pmax when their cadence fell below 70 rev/min for more than 30 s. This criterion was repeated for further reductions to 70%Pmax and 60%Pmax, respectively. Participants continued cycling with the alternating 2 min periods at each work interval at 50%Pmax with 80 rev/min until they could no longer maintain the required cadence over a minute. Heart rate was monitored every minute. Height, weight, body fat, and body mass index (BMI) were measured using a body composition analyzer (Inbody 720, Biospace, Korea) before and immediately after the glycogen depletion session. Blood lactate was measured before and after the endurance capacity test using a lactate scout analyzer (SensLab, LSSY-170407-E, Germany). VO_2_, VCO_2_ and RER (Vmax Sensor Medics Metabolic, SensorMedics®, USA) were measured continuously during the endurance capacity test.

### Recovery period

After completing the glycogen depletion session, participants rested for 2 h in the laboratory. The volume of LFM provided was calculated such that participants received 1 g of CHO/kg BM [5,6] during the recovery period. For example, if the participant had a body mass of 66 kg, he would receive 1320 ml of LFM (12.5 g CHO/250 ml LFM). By selecting a commercially available sports drink with a caloric content similar to LFM, both volume and caloric content of test drinks ingested during the SPD and LFM trials were matched. An equal volume of water was provided during the WT trial. Each test drink was administered in three aliquots during the recovery period: 50% at 0 min, 25% at 30 min and the remaining 25% at 60 min. [14,15].

### Endurance capacity test

Following the recovery period, participants completed a standardized warm-up (2 min at 60 rev/min, workload of 0.5 kp) before embarking on the endurance capacity test at 80 rev/min at 70%VO_2_max. The test was terminated when the participant’s cadence fell below 70 rev/min for more than 30 s twice [5]. VO_2_, VCO_2_, and RER were measured during the endurance capacity test. Heart rate was recorded continuously. Time to exhaustion was measured. Rating of perceived exertion (RPE) were measured every 5 min [16]. Body weight and blood lactate were measured at the start and immediately at the end of the trial.

### Data analysis

Statistical analyses were performed using SPSS (IBM® Corp, SPSS®Statistics Version 21, USA). One-way repeated measures analysis of variance (ANOVA) was used to compare all variables (e.g. heart rate, oxygen uptake and cycle time to exhaustion) between trials (i.e. WT, SPD and LFM). Data were presented as mean ± standard deviation (SD). Alpha was set *a priori* at 0.05.

## Results

Physical and physiological characteristics of participants are shown in Table 1. Participants were similarly euhydrated (urine specific gravity; WT: 1.014 ± 0.004, SPD: 1.014 ± 0.004, LFM: 1.020 ± 0.003; p = 0.57) and had similar absolute (WT: 2019 ± 573, SPD: 1722 ± 487, LFM: 2113 ± 389 kcal/day; p = 0.95) and relative caloric intakes (WT: 30 ± 3, SPD: 26 ± 8, LFM: 31 ± 6 kcal/kg/day; p = 0.39) prior to undertaking the experimental trials.

Heart rate was similar across all three experimental trials (Table 3). Whilst oxygen consumption and RER were similar during all three glycogen depletion sessions, oxygen uptake was lower in LFM (p < 0.05) during the endurance capacity test than in SPD and WT. In addition, RER was lower in WT than SPD during the endurance capacity test (p < 0.05).

**Table 3:** **Physiological responses during each trial**

	**Water**	**Sports drink**	**Lactose-free milk**
**Glycogen depletion session**			
Heart rate (beats/min)	136 ± 7	139 ± 6	135 ± 7
VO_2_ (ml/min/kg)	38 ± 1	38 ± 1	36 ± 1
VCO_2_ (ml/min/kg)	42 ± 2	43 ± 2	41 ± 2
RER	1.11 ± 0.02	1.11 ± 0.01	1.12 ± 0.02
**Endurance performance test**			
Heart rate (beats/min)	153 ± 13	153 ± 13	152 ± 13
VO_2_ (ml/min/kg)	35 ± 4	34 ± 4	30 ± 4*#
VCO_2_ (ml/min/kg)	33 ± 5	34 ± 4	29 ± 4*#
RER	0.95 ± 0.05	0.99 ± 0.05*	0.97 ± 0.03
RPE	16 ± 4	16 ± 4	14 ± 5

*p < 0.05 compared with water; # p < 0.05 compared with sports drink.

**Figure 1:**
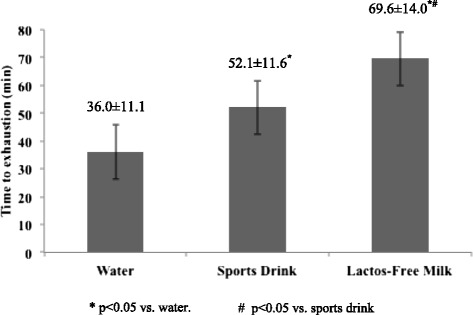
**Endurance capacity between trials.** *p < 0.05 vs. water. #p < 0.05 vs. sports drink.

Time to exhaustion was greatest in LFM (p < 0.05), followed by SPD and WT (Figure 1). The longer exercise duration in LFM also elicited the greatest body mass loss compared to the other two trials (LFM 1.0 ± 0.3 kg; SPD 0.8 ± 0.3 kg; WT 0.6 ± 0.3 kg; p < 0.05). Blood lactate was higher after the endurance capacity test with WT (2.4 ± 1.9 mmol/dl) than SPD (1.0 ± 0.7 mmol/dl; p < 0.05), but neither was significantly different in comparison to LFM (1.7 ± 1.4 mmol/dl; p > 0.05).

## Discussion

Our study is the first to demonstrate the ergogenic effects of ingesting lactose-free milk on endurance cycling capacity in young adults. The ingestion of lactose-free milk during recovery after glycogen depleting exercise almost doubled (93% increase) the subsequent cycle time to exhaustion in comparison with water, and further extended exercise duration by 34% compared to sports drink. Despite identical workloads during the endurance capacity test with all three drinks, the oxygen uptake and concomitant expired carbon dioxide during exercise was lower after the ingestion of lactose-free milk compared to water and sports drink. This may indicate that the ingestion of lactose-free milk during recovery enhances metabolic efficiency during the subsequent exercise bout.

Although muscle glycogen resynthesis was not measured in the present study, this may have been enhanced with the ingestion of lactose-free milk compared to the isocaloric sports drink [9,17]. Previous work has generally shown a greater muscle glycogen resynthesis with carbohydrate-protein drinks compared to carbohydrate-only drinks [8,18], although mixed results exist [19]. The interpretation of these results, however, is limited by a higher caloric content in the carbohydrate-protein drinks than the carbohydrate-only drinks. Whilst it has been suggested that a greater carbohydrate intake may enhance glycogen resynthesis to match that after carbohydrate-protein intake [20], other comparisons of isocaloric carbohydrate-only and carbohydrate-protein drinks nevertheless demonstrate a more efficient replenishment of muscle glycogen after carbohydrate-protein intake [9,17]. Moreover, drinks containing too much carbohydrate (8–10%), similar to the sports drink in the present study, may also delay gastric emptying and fluid absorption [15]. This could result in reduced muscle and liver glycogen stores. Taken together, it is thus possible that muscle glycogen resynthesis was enhanced with ingestion of lactose-free milk compared to sports drink (and water) in the present study. An improved glycogen repletion would enable a better metabolic efficiency during the cycle ride to exhaustion after ingestion of lactose-free milk and explain, in part, the lower oxygen uptake required to sustain exercise and the resultant extension in exercise duration [8].

Differences in the type of carbohydrate in lactose-free milk compared to sports drink could also have contributed to the lower oxygen consumption observed during the cycle ride to exhaustion in the present study. Whilst SPD contained 6% glucose and 4% sucrose, LFM consisted of 2.5% glucose and 2.5% galactose. Exogenous glucose oxidation during exercise has a maximum of 1.0–1.1 g/min, whereas galactose utilization is limited to ~0.4 g/min [14]. This lower oxidation rate of galactose compared to glucose and sucrose [21] is due to the conversion of glucose in the liver, before subsequent utilization by the skeletal muscles. The ingested galactose may also have been synthesized to form glycogen during the recovery period. It is thus possible that the galactose in lactose-free milk slowed the oxidation process, resulting in the lowered oxygen consumption and carbon dioxide production observed [4].

A reduction in muscle damage (i.e. creatine phosphokinase levels [18]) with lactose-free milk ingestion is another likely candidate to explain the physiological differences observed during the cycle ride to exhaustion in the present study. This may be achieved via a reduced rate of protein breakdown (i.e. improved whole body net protein balance [22]) and greater myofibrillar muscle protein synthesis through p70S6K, downstream of mTOR [23], following the ingestion of protein after exercise. Whether the performance benefits associated with lactose-free milk ingestion in the present study are also relevant to a more aerobically fit population (e.g. VO2peak 65 ± 7 mL/min/kg [24]) is unclear, and requires further investigation.

Whilst the underlying mechanism(s) for the lower oxygen consumption and carbon dioxide production with LFM cannot be elucidated from the present study, our results nonetheless clearly demonstrate the efficacy of LFM as a recovery drink to enhance subsequent exercise performance. The extended time to exhaustion after LFM ingestion observed in the present study is in agreement with previous work that have investigated chocolate milk as a recovery drink [5,6]. Those studies have reported that the ingestion of chocolate milk as a recovery drink results in at least comparable, if not better, subsequent exercise performance compared to other commercially available fluid- and carbohydrate-replacement drinks [5,6].

Further work is necessary to determine the mechanism(s) underlying the extended endurance capacity duration after lactose-free milk ingestion, compared to an isocaloric and isovolumic sports drink. In the present study, participants could not be blinded to the test drink and this may have psychologically affected them during the cycle ride to exhaustion. Whilst the efficacy of lactose-free milk as a recovery drink is clear, the product used the present study was manufactured in-house and fortified with sodium and potassium. This is a key limitation for the immediate applicability of our results to the lactose intolerant population, as the lactose-free milk investigated in the present study is not currently commercially available.

## Conclusion

This study demonstrates an increased cycle time to exhaustion after ingesting lactose-free milk as a recovery drink, as compared to water and sports drink. The extended exercise duration may be explained by a greater metabolic efficiency, as the amount of oxygen consumed and carbon dioxide produced were both reduced with lactose-free milk. Consequently, lactose-free milk may be appropriate as a recovery drink for the general population and can be used as a substitute for normal milk in lactose intolerant individuals.
